# Compatibility and Fidelity of Mirror-Image Thymidine in Transcription Events by T7 RNA Polymerase

**DOI:** 10.1016/j.omtn.2020.06.023

**Published:** 2020-06-27

**Authors:** Qingju Liu, Yongqi Ke, Yuhe Kan, Xinjing Tang, Xiangjun Li, Yujian He, Li Wu

**Affiliations:** 1 School of Chemical Sciences, University of Chinese Academy of Sciences, Beijing 100049, China; 2 State Key Laboratory of Natural and Biomimetic Drugs, School of Pharmaceutical Sciences, Peking University, Beijing 100191, China

**Keywords:** T7 RNA polymerase, transcription, l-thymidine, mismatch, self-repair

## Abstract

Due to highly enzymatic d-stereoselectivity, l-nucleotides (l-2′-deoxynucleoside 5′-triphosphates [l-dNTPs]) are not natural targets of polymerases. In this study, we synthesized series of l-thymidine (l-T)-modified DNA strands and evaluated the processivity of nucleotide incorporation for transcription by T7 RNA polymerase (RNAP) with an l-T-containing template. When single l-T was introduced into the transcribed region, transcription proceeded to afford the full-length transcript with different efficiencies. However, introduction of l-T into the non-transcribed region did not exhibit a noticeable change in the transcription efficiency. Surprisingly, when two consecutive or internal l-Ts were introduced into the transcribed region, no transcripts were detected. Compared to natural template, significant lags in NTP incorporation into the template T+4/N and T+7/N (where the number corresponds to the site of l-T position, and + means downstream of the transcribed region) were detected by kinetic analysis. Furthermore, affinity of template T+4/N was almost the same with T/N, whereas affinity of T+7/N was apparently increased. Furthermore, no mismatch opposite to l-T in the template was detected in transcription reactions via gel fidelity analysis. These results demonstrate the effects of chiral l-T in DNA on the efficiency and fidelity of RNA transcription mediated by T7 RNAP, which provides important knowledge about how mirror-image thymidine perturbs the flow of genetic information during RNA transcription and development of diseases caused by gene mutation.

## Graphical Abstract

**Figure:**
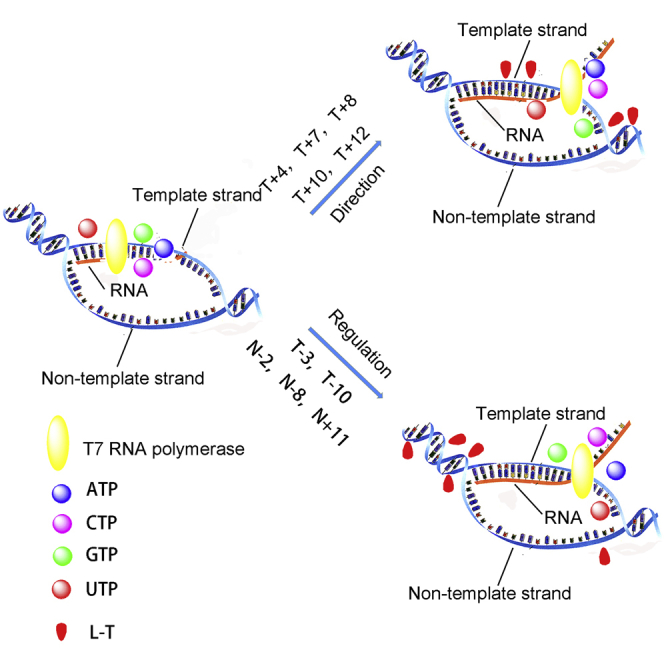

## Teaser

Transcription proceeded to afford the full-length transcript with different efficiencies when single l-T was introduced into templates. When two consecutive or internal l-Ts were introduced into the transcribed region, no transcripts were detected. No mismatch opposite to l-T in the template was detected.

## Introduction

Chirality of the repeating units of polymers is an essential factor for the formation of the higher-order structure and function of diverse living organisms.^1^ For the origin of life on Earth, four types of nucleosides (A, G, C, and T(U)) are initially acknowledged to construct the building blocks of living macromolecules. Despite that both isomers exist in nature, universal organisms on Earth utilize only the d-nucleosides as building blocks of DNA chains. However, recent investigations report that the non-natural nucleobase pairs in duplex DNA can be efficiently duplicated in PCR reactions,^2^ transcribed to RNA,^3^^,^^4^ and used to encode to proteins with non-standard amino acids,^4^^,^^5^ paving the way for the development of a semisynthetic organism.

Possibly, damage of chirality of DNA occurs owing to the presence of l-nucleoside analogs (lamivudine, emtricitabine, and telbivudine) in living cells as a new class of antiviral drugs.^6^^,^^7^ Substantial investigations indicate the incorporation of chiral nucleotide (nt) analogs during DNA synthesis because of impairment or loss of d-stereoselectivity^8^, ^9^, ^10^ in non-enzymatic^11^, ^12^, ^13^ or enzymatic^9^^,^^14^, ^15^, ^16^ template-directed polymerizations. To the best of our knowledge, little is known about the follow-up effects on the evolution of life after incorporation of mirror-image nucleosides into a DNA strand. Recently, we reported that natural nucleotides could be incorporated into the growing chain opposite of the l-thymidine (l-T) unit in the DNA template by B family DNA polymerases (Therminator, Vent [exo^−^], and Deep Vent [exo^−^] DNA polymerase) and elongate the primer with self-repair.^17^ Based on the results of primer extension, further investigation on the role of mirror-image thymidine in bypassing these lesions in RNA transcription reactions was necessary.

Transcription is a continuous process of synthesis of an RNA copy from the DNA templates, catalyzed by RNA polymerase (RNAP) with high fidelity.^18^, ^19^, ^20^ T7 RNAP is a favorable bacteriophage RNAP. Containing a highly specific promoter recognition site and a nascent RNA-binding domain in its catalytic domain, T7 RNAP works self-sufficiently and drives transcription strongly without transcription factors.^20^, ^21^, ^22^ T7 RNAP exhibits an efficient elongation rate (about 130 nt/s) and displays a relatively high level of template-dependent transcriptional infidelity (2 × 10^−6^ error rate).^23^ The ability to distinguish between correct and incorrect nucleotides is significant for its high efficiency and fidelity control of transcription.^24^ In general, it is assumed that the correct substrate binds with higher affinity. However, many researchers have studied the site-specific incorporation of emissive pyrimidine analogs,^25^ fluorescent ribonucleotide analog 1,3-diaza-2-oxophenothiazine-ribose-5′-triphosphate (tCTP),^26^ 2′-methylseleno-2′-deoxyuridine 5′-triphosphate,^27^ nucleotides with 2′-modified ribose moieties,^28^ and modified uridines^29^^,^^30^ to effectively generate labeling RNA strands by T7 RNAP. Previous results have focused on incorporation of mirror-image nucleoside 5′-triphosphate (NTP) as a substrate by RNAP.^9^^,^^14^ It is of our interest to evaluate the compatibility and fidelity of T7 RNAP with mirror-image nucleotide-containing templates.

Although DNA replication studies with A and B family DNA polymerases have been carried out for l-T insertion,^17^ our transcriptional systems were used to estimate the degrees to which the lesion impedes RNA transcription and whether it induces mutations. In this study, we have introduced l-T into different sites of template strands and non-template strands, and then examined the processivity of transcription with the modified templates catalyzed by T7 RNAP. Upon introduction of l-T into non-transcribed regions of template or non-template, transcription was able to efficiently achieve full-length transcripts. When l-T was introduced into a transcribed region of templates, T7 RNAP incorporated NTP with distinct catalytic efficiencies. Notably, transcription catalyzed by T7 RNAP was aborted when two consecutive or internal l-Ts were introduced into a transcribed region. In addition, T7 RNAP incorporated NTP with high catalytic efficiency along the mirror-image-containing template with no mismatch. Moreover, structural insights into the NTP incorporation clarified the detailed mechanism by combining with their transcriptional activities and kinetic analysis.

## Results and Discussion

### Introduction of l-T into DNA Strands for Regulation of Transcription

**Figure 1:**
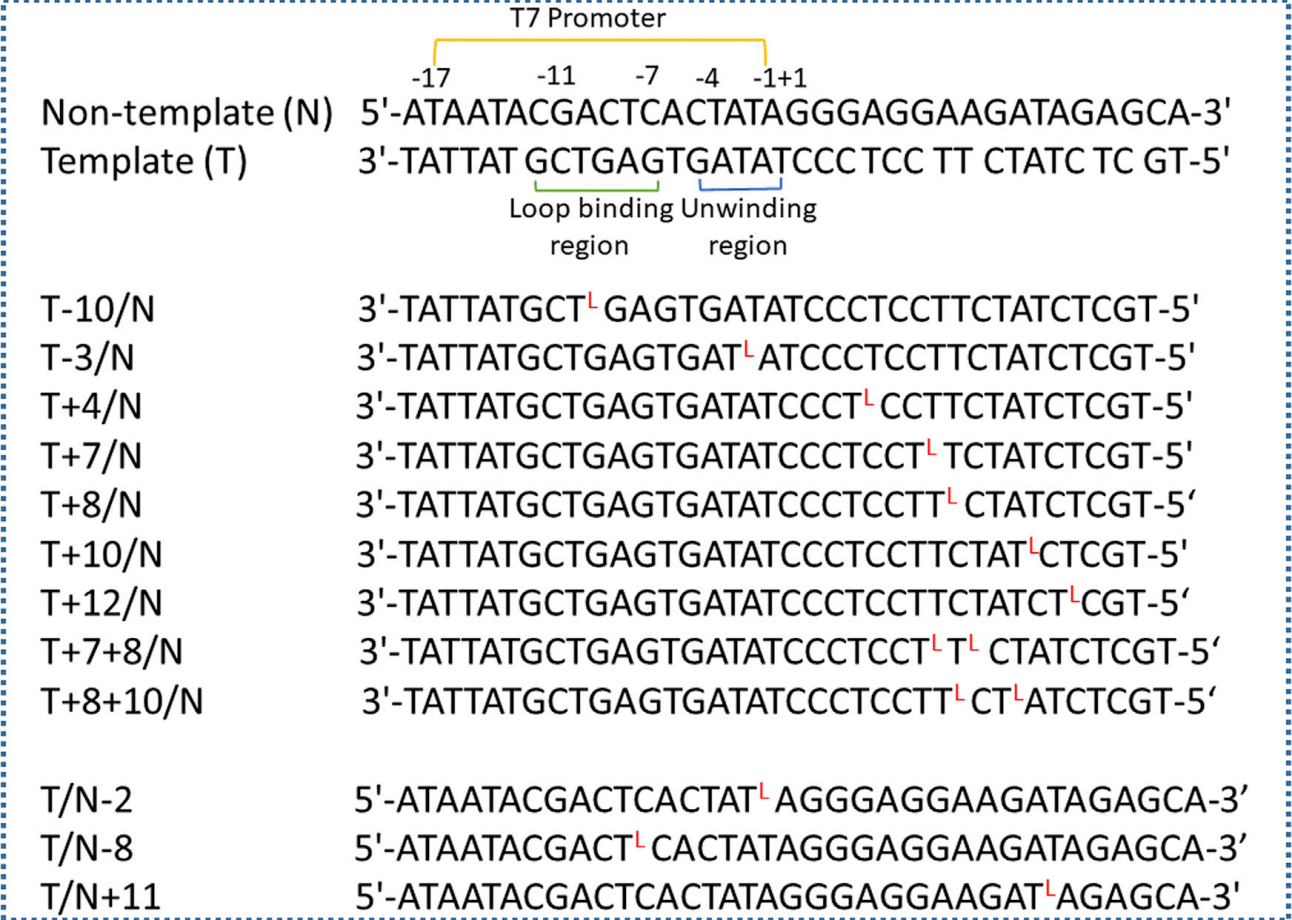
Sequences of Templates Used in the Present Study

T7 promoter includes two functional regions, that is, an RNAP recognition region and an unwinding region.^31^^,^^32^ To test directly the efficiency of transcription with l-T-containing templates by T7 RNAP, we synthesized the l-T phosphoramidite to introduce the l-T unit into either the non-template strand (N) or the template strand (T) at different sites as shown in Figure 1.

### Transcription with Templates Containing a Single l-T

**Figure 2:**
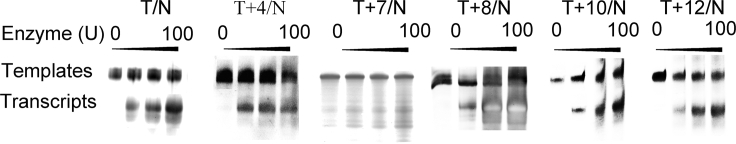
PAGE of Products of Transcripts from Templates Containing l-T at Different Positions of the Transcribed Region in the Template Strand Transcription reactions were carried out using T7 RNAP on templates T/N, T+4/N, T+7/N, T+8/N, T+10/N, and T+12/N. The amounts of T7 RNAP of four lanes with the same template were 0, 25, 50, and 100 U/mL, respectively. The template concentration of each lane was 1 μM. The positions of the migration of the DNA templates and the RNA products were marked on the left on the gel. The DNA template used in each reaction is indicated at the top of each lane.

To evaluate the effect of l-T on the efficiency of transcription catalyzed by T7 RNAP, a transcription reaction with template containing l-T at positions +4, +7, +8, +10, and +12 of the transcribed region (T+4/N, T+7/N, T+8/N, T+10/N, and T+12/N) was performed. A transcription reaction was carried out in the presence of all four natural NTPs. As shown in Figure 2 and Table S2, with a fully standard system, the results were evidently different. Transcription of normal templates gave a full-length product without pausing (template T/N); the correct full-length product was also formed and transcription efficiencies were similar when l-T was introduced into the transcribed region (template T+4/N, T+8/N, T+10/N, and T+12/N). The transcription products from template T+7/N were analyzed on denaturing gel. Full-length products as well as aborted transcripts could be visualized in the gel, indicating that transcription of the template T+7/N was less efficient than for other templates. These data suggest that T7 RNAP possessed almost the same catalytic properties during RNA synthesis with these l-T-containing templates except for T-7/N.

### Transcription with Templates Containing l-T in a Non-transcribed Region of Template Strand

**Figure 3:**
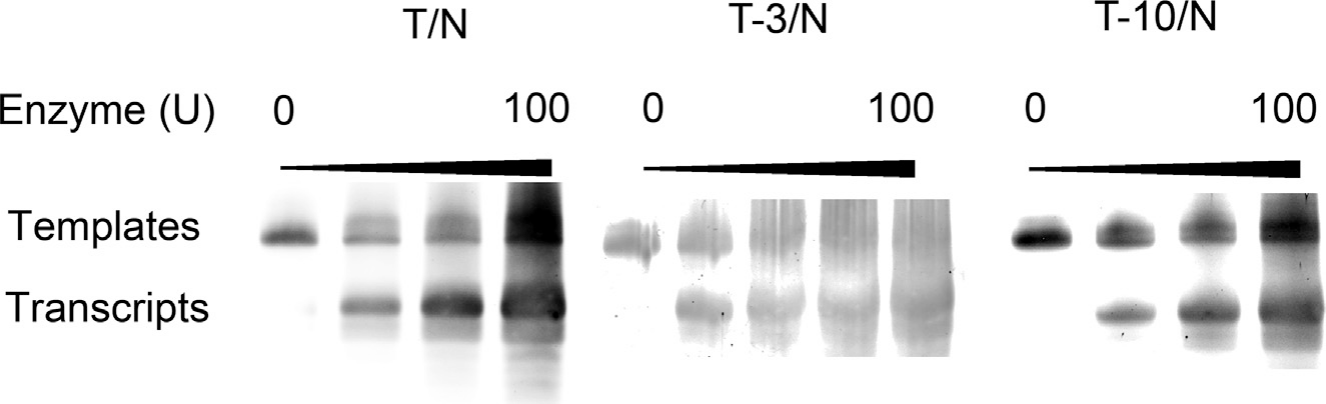
PAGE of Transcripts from Templates Containing l-T at Different Positions of the Non-transcribed Region in the Template Strand Transcription reactions were carried out using T7 RNAP on templates T/N, T−3/N, and T−10/N. The amounts of T7 RNAP of four lanes with the same template were 0, 25, 50, and 100 U/mL, respectively. The template concentration of each lane was 1 μM. The positions of the migration of the DNA templates and the RNA products were marked on the left on the gel. The DNA template used in each reaction is indicated at the top of each lane.

Therefore, an investigation of transcription reaction with templates containing l-T in the T7 promoter was conducted. Several transcription experiments were performed using the templates modified with l-T at position −3 and −10 (template T−3/N and T−10/N) in the T7 promoter in the presence of T7 RNAP. As shown in Figure 3 and Table S3, the uppermost bands represented DNA templates and the lower bands indicated the full-length transcripts. The full-length runoff transcripts observed in the presence of T−3/N and T−10/N appear qualitatively similar to that observed using the normal template T/N. No remarkable increase or decrease in transcription efficiencies occurred. These observations suggest that introduction of l-T into a non-transcribed region of template strand has no impact on the process of transcription.

### Transcription with Templates Containing l-T at Different Positions of Non-template

**Figure 4:**
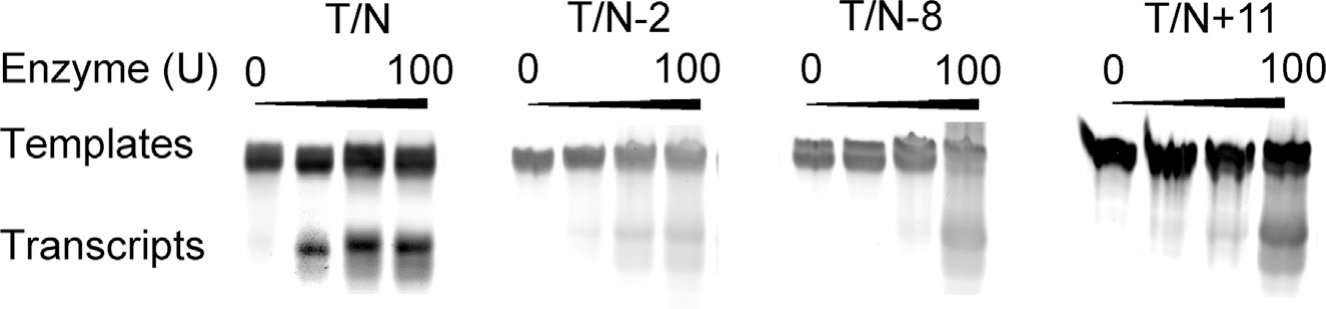
PAGE of Transcripts from Templates Containing l-T at Different Positions of the Non-template Strand Transcription reactions were carried out using T7 RNAP on templates T/N, T/N−2, T/N−8, and T/N+11. The amounts of T7 RNAP of four lanes with the same template were 0, 25, 50, and 100 U/mL, respectively. The template concentration of each lane was 1 μM. The positions of the migration of the DNA templates and the RNA products were marked on the left on the gel. The DNA template used in each reaction is indicated at the top of each lane.

Additionally, non-template is essential for the transcriptional reaction. To further understand the role of l-T in transcription, templates T associated with non-template N strand containing l-T units at positions −2, −8, and +11 were utilized to test the regulation of transcription in the presence of T7 RNAP. Strikingly, similar results were obtained for introduction of l-T in the non-template strand by comparing it with natural DNA template. In the case of template T associated with the non-template strand containing l-T at positions −2, −8 and +11 (T/N−2, T/N−8 and T/N+11), the same amounts of 17-nt transcripts were detected, indicating that the introduction of l-T at different sites of non-template strand did not result in a sharp decrease or increase in the yields of transcripts (Figure 4; Table S4). This phenomenon may reflect unaffected transcription initiation at the promoter owing to an insignificant increase in steric hindrances.

### Transcription with Templates Containing Two l-Ts

**Figure 5:**
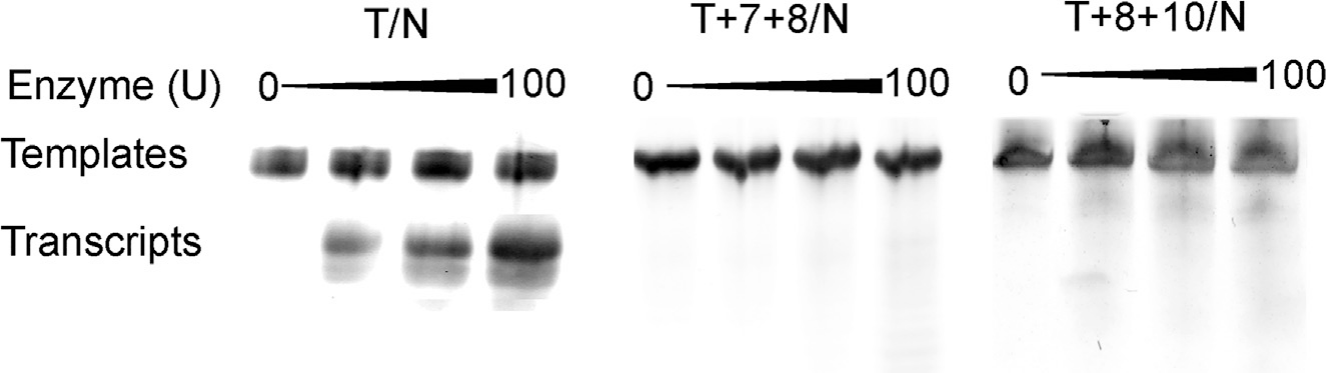
PAGE of Transcripts from Templates Containing Two l-Ts at Different Positions of the Transcribed Region in the Template Strand Transcription reactions were carried out using T7 RNAP on templates T/N, T+7+8/N, and T+8+10/N. The positions of the migration of the DNA templates and the RNA products were marked on the left on the gel. The DNA template used in each reaction is indicated at the top of each lane.

To investigate the synergistic effect of multiple l-Ts on transcriptional efficiency, several transcription experiments were performed with template containing two consecutive l-Ts (T+7+8/N) and two interval l-Ts (T+8+10/N). Extraordinarily, with modification of two consecutive l-Ts at positions +7 and +8 in the transcribed region, no noticeable bands of any transcriptional products were detected, indicating that incorporation of NTP was suppressed in the synthesis. Similarly, transcription with templates containing two interval l-Ts (T+8+10/N) was unable to proceed, and no detectable transcription products were found in the gel (Figure 5; Table S5). Although the structural differences induced by a single l-T-modified template are able to be recognized by T7 RNAP, introduction of two l-Ts in the transcribed region results in accumulated conformation change and increases steric hindrances during the transcription process, which may suppress the transcription reaction.

### Transcription Kinetics Analysis

**Figure 6:**
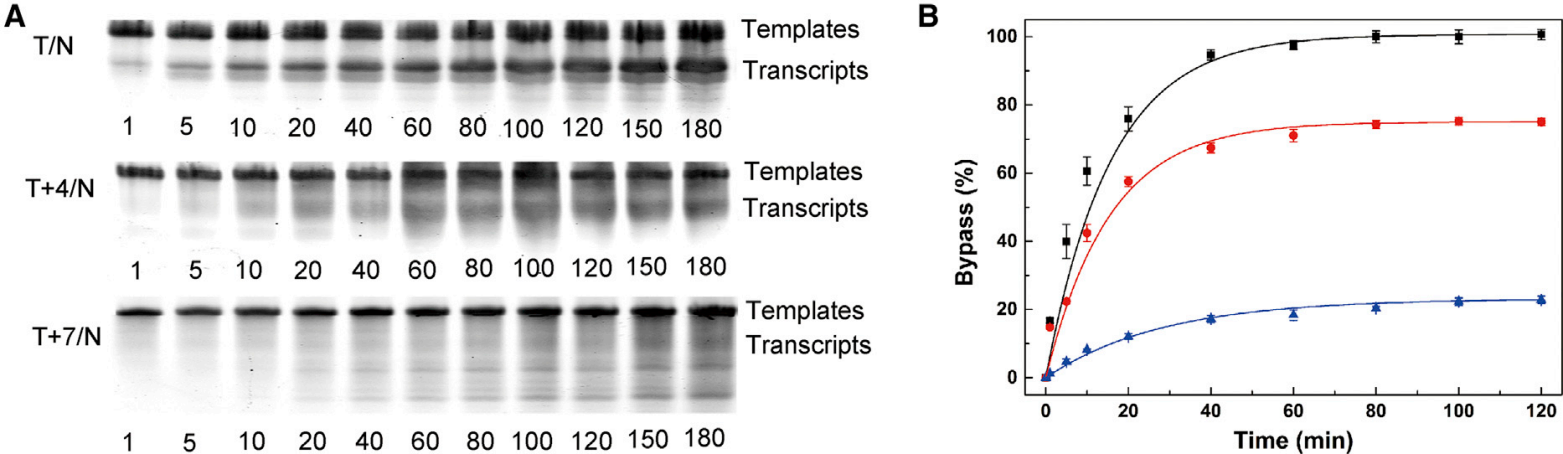
Running Start Assays of Transcription Reactions Conducted with Three Templates (T/N, T+4/N, and T+7/N) by T7 RNAP (A) Analysis of transcripts of the three templates at various times on denaturing gels. (B) Kinetic curves of transcripts from different templates. A plot of the amount of 17-mer versus time for reactions carried out on the three templates and fit to A(1 − e^−kobs*t*^) is shown, where A is the amount of 17-mer transcripts at long time points, and k_obs_ is the time constant for productive transcription initiation (as measured by the appearance of the 17-mer transcripts). ▪, T/N; ●, T+4/N; ▲, T+7/N.

Because T7 RNAP can incorporate NTPs into transcripts directed by templates containing a single l-T, a running start assay was determined to confirm the effects of l-T on *in vitro* DNA transcription. Figure 6A showed amounts of the transcript of natural and l-T-modified template (T/N, T+4/N, T+7/N) in the presence of all four NTPs (0.5 mM) and T7 RNAP (5 U/μL) for various times, respectively. Evidently, transcripts directed by a natural template were comparatively more abundant than those of template T+4/N. In addition, nucleotide incorporation into RNA products directed by template T+4/N became relatively slow. Similar to template T+4/N, transcription directed by template T+7/N showed a more serious lag in incorporating NTPs compared to a natural DNA template. Moreover, abortive and intermediate products were also observed with template T+7/N.

Quantitative analyses of running start assays were determined by full-length production formation (l-T, bypass %) versus reaction time (Figure 6B).^33^ RNA synthesis for the three transcription reactions with T/N, T+4/N, and T+7/N show a constant level within 60 min. The kinetic parameters (observation rate constant [k_obs_]) of transcription were obtained by A(1 − e^−k^^obs^^*t*^) (where A is the amplitude of the reaction and *t* is the time in seconds), and k_obs_ values for nucleotide insertions opposite l-T for template T+4/N and T+7/N were 1.05 × 10^−2^ s^−1^ and 0.5 × 10^−2^ s^−1^, respectively, versus 1.19 × 10^−2^ s^−1^ for d-T. The least efficient transcription (T+7/N) led to synthesis of a 2.38-fold (1.19 × 10^−2^/0.5 × 10^−2^) lower amount of runoff RNA than for the most efficient transcription (T/N).

**Figure 7:**
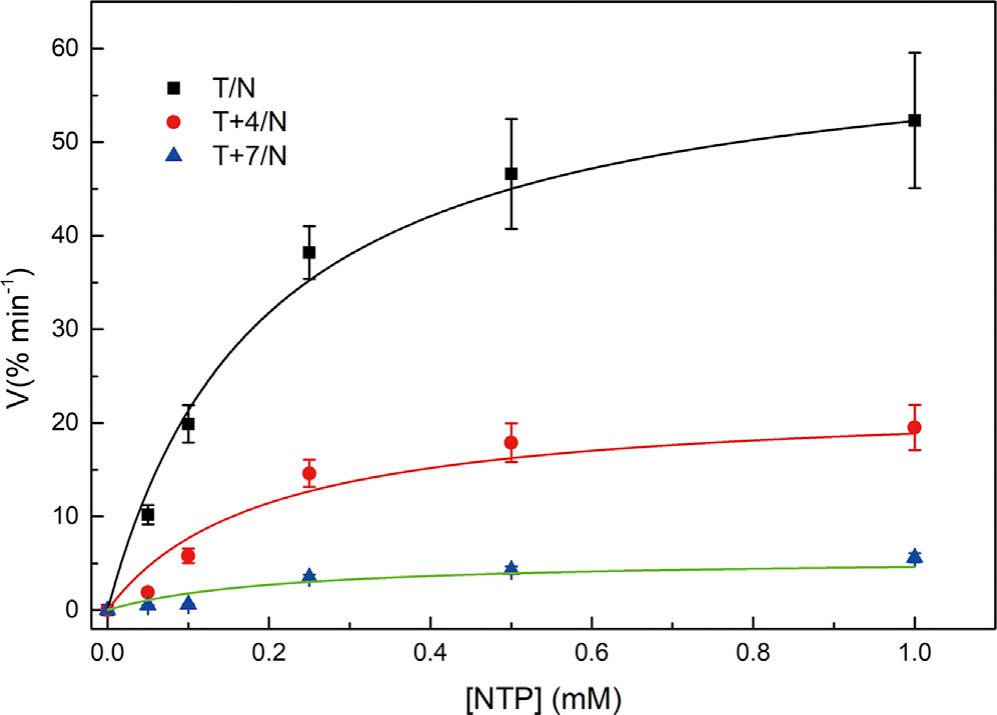
Michaelis-Menten Plots of the Transcription Reaction (Transcript Formation) by T7 RNAP as a Function of the Concentration of NTP with Templates ▪, T/N; ●, T+4/N; ▲, T+7/N. The concentration of T7 RNAP was kept at 5 U/μL.

As described above, transcription activity was higher with normal templates than with the l-T-containing templates. Subsequently, kinetic parameters (K_m_ [Michaelis-Menten constant] and k_cat_ [catalytic constant for the conversion of substrate into product]) were performed with T+4/N and T+7/N as well as the natural template at various NTP concentrations, and the gel analyses are shown in Figure S6. Then, the rate of transcription (v, μM·min^−1^) was plotted as a function of NTP concentration, and the data were fit by Michaelis-Menten plots of transcription rate as a function of the concentration of NTP (OriginLab).^31^^,^^34^ Interestingly, the curves of these two templates were entirely different (Figure 7), indicating that introduction of an l-T into templates T+4/N and T+7/N played a different role in transcription. From these plots, K_m_ and k_cat_ of the two templates could be calculated as listed in Table 1. Comparison of T+4/N with T/N revealed that K_m_ for T+4/N was almost the same with that for T/N (K_m_s for T/N and T+4/N were 0.192 and 0.193 mM, respectively), whereas K_m_ for T+7/N was 0.214 mM, demonstrating that introduction of l-T at position +7 of the transcribed region strongly interfered with the binding affinity of RNAP. Moreover, compared with templates T/N and T+4/N, the K_m_ did not change, but the k_cat_ significantly changed. In contrast, k_cat_ values of transcription with T+7/N as the template were remarkably dropped compared to that of the normal template (1.12 versus 12.46 min^−1^), suggesting a noticeable decrease in the transcript formation rate with template T+7/N. Considering that changes in kinetic parameters are affected by structural changes of template, substrate, or the polymerase,^34^ the results suggest that template T+7/N induces much larger disordering than does T+4/N.

**Table 1:** Kinetic Parameters for T/N, T+4/N, and T+7/N Templates Determined from the Michaelis-Menten Plots

Template	K_m_ (mM)	k_cat_ (min^−1^)	k_cat_/K_m_ (mM^−1^ min^−1^)
T/N	0.192	12.46	64.9
T+4/N	0.193	4.5	23.3
T+7/N	0.214	1.12	5.2

### Fidelity of Nucleotide Incorporation Opposite l-T in the Template

In the RNA synthesis, the selection of nucleotide substrate is based on the Watson-Crick base pairing between a template DNA nucleotide and the incoming NTP with the aid of RNAP.^35^ T7 RNAP achieves an error rate of ~2 × 10^−6^ in elongation,^18^^,^^23^ and the transcription accuracy or fidelity control relies fully on the nucleotide selectivity prior to the nucleotide incorporation.^35^ Previous studies have shown that l-nucleotide residue in heterochiral duplexes retains its selectivity toward the complementary d-nucleotide residue.^17^^,^^36^ Considering that T7 RNAP can incorporate NTPs opposite l-T in the templates into transcripts with different efficiency, we therefore checked the fidelity of the T7 RNAP when it encountered l-T-containing templates.

**Figure 8:**
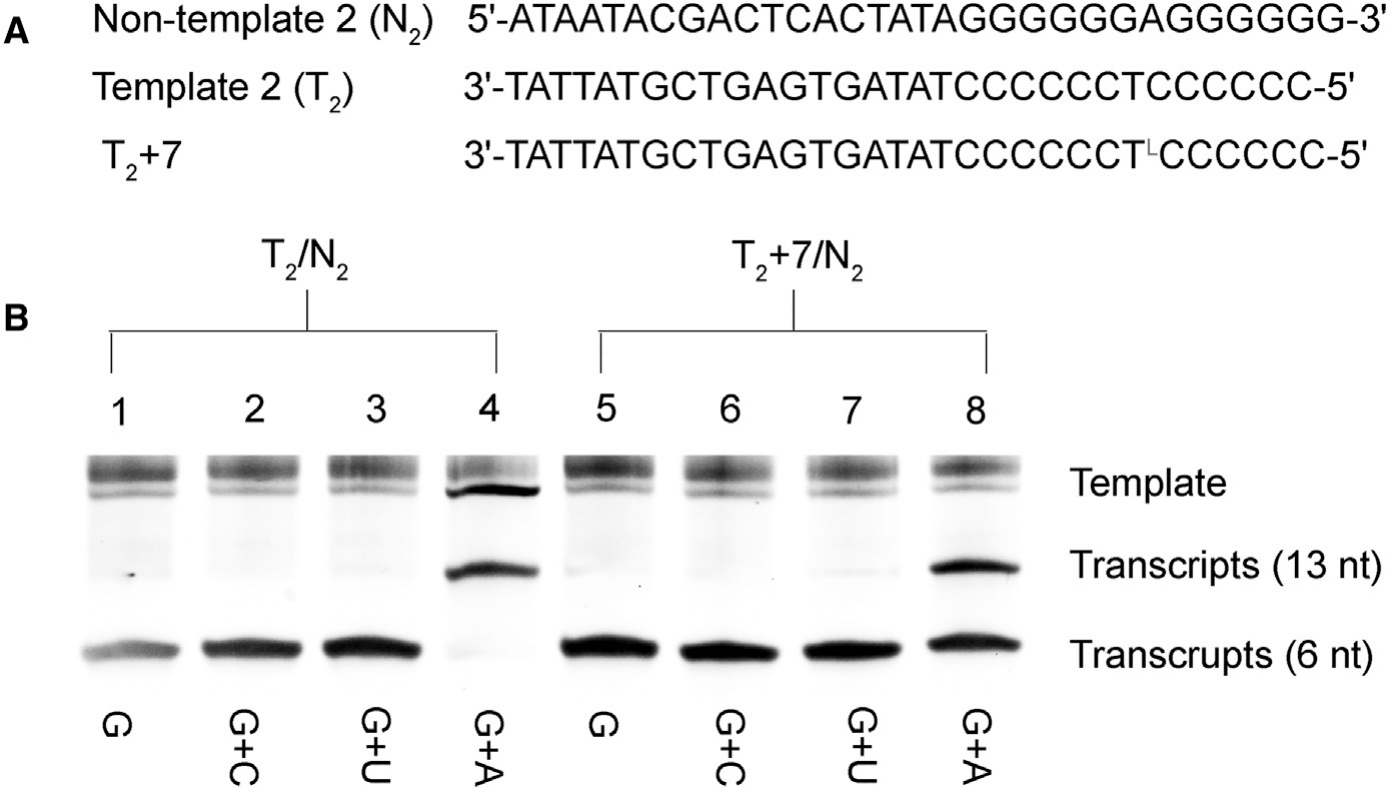
Fidelity of Incorporation of NTP Opposite l-T in a Natural Template (T_2_/N_2_ and T_2_+7/N_2_) in a Transcription Reaction Catalyzed by T7 RNAP Sequences of templates (A) and PAGE of transcripts (B) in the fidelity analysis. The NTP added is indicated on the bottom of each lane. Transcription experiments were performed with a standard reaction system except that the NTP mixtures were substituted to a single nucleotide.

Considering that T7 RNAP exhibits a strong bias for guanosine triphosphate (GTP) as the initiating nucleotide,^37^ we therefore designed templates that contained six consecutive cytidine units placed at positions from +1 to +7 in the transcribed region (Figure 8A). With the standard transcription system, gel fidelity analysis was performed with GTP, GTP+cytidine triphosphate (CTP), GTP+uridine triphosphate (UTP), or GTP+ATP in independent reaction tubes with natural templates T_2_/N_2_ or l-T-modified template T_2_+7/N_2_. As described in Figure 8B and Table S6, only the addition of GTP+ATP into natural templates T_2_/N_2_ can obtain the full-length transcription product, and the transcription was terminated when encountering the d-T in the templates. Similarly, the band of full-length transcripts was clearly observed in the gel in the presence of GTP+ATP with T_2_+7/N_2_ as template. However, compared to T_2_/N_2_, the amounts of products were remarkably smaller, although an obvious band of aborted products (6 nt) was detected, indicating the much lower transcription efficiency for T_2_+7/N_2_. When GTP, GTP+CTP, or GTP+UTP was added into the reaction system, only the aborted transcripts were detected and full-length products were not found in the gel, suggesting that the T7 RNAP cannot incorporate GTP, CTP, or UTP opposite l-T in the template to continue the RNA synthesis process by T7 RNAP. Only ATP could be incorporated efficiently with the complementary nucleotides (d-T or l-T) on the template, without significant mismatch having been observed.

### Structural Insight into NTP Incorporation Opposite l-T in the Template

We systematically investigated the compatibility and fidelity of l-T in transcription reactions by T7 RNAP, and our results led to several important findings. First, we observed that single l-T can be bypassed in all transcription reactions to afford the full-length transcript with different efficiencies. Introduction of l-T into the non-transcribed region of template or non-template does not result in remarkable change in transcription efficiency. However, l-T at the +4 and +7 positions of the transcription region inhibited RNA transcriptions, and the incorporation inhibited at position +7 was much stronger than that at position +4, suggesting that the l-Ts at the +7 position were not much better tolerated in RNAP transcription elongation. According to our results from Gaussian calculations, the Gibbs free energy for template T+7/N (l-T) binding with RNAP (−300.9 kcal/mol) is higher than that for template T+7/N (d-T) (−309.4 kcal/mol) (Table S7). Second, we found that transcription of template containing two consecutive (T+7+8/N) and internal l-Ts (T+8+10/N) is suppressed by T7 RNAP. Third, our results demonstrated that the nucleotide insertion opposite l-Ts is specific, where only the T-A base pair was formed for T7 RNAP.

T7 RNAP exhibits powerful transcription efficiency *in vitro*.^29^ As an important stage of gene expression and regulation,^38^ transcription of DNA by T7 RNAP takes place in three phases, that is, initiation, elongation, and termination.^26^ T7 RNAP initiates RNA synthesis at promoter sequences that are conserved from positions −17 to +6 relative to the start site of transcription.^39^ The N-terminal promoter binding domain of T7 RNAP closely connects with a specific promoter sequence, melting the DNA duplex, and it directs the template strand into the catalytic site of transcription.^40^^,^^41^ Abortive products fewer than 8–10 bases frequently are obtained in this phase of RNA synthesis (Figure 2).^26^ Subsequently, when the nascent RNA achieves 8–12 nt, T7 RNAP peels away from the promoter sequence by a conformational change and enters the highly processive and efficient elongation mode.^26^^,^^42^ When the enzyme encounters a terminator signal or when it reaches the end of template, transcription terminates.

**Figure 9:**
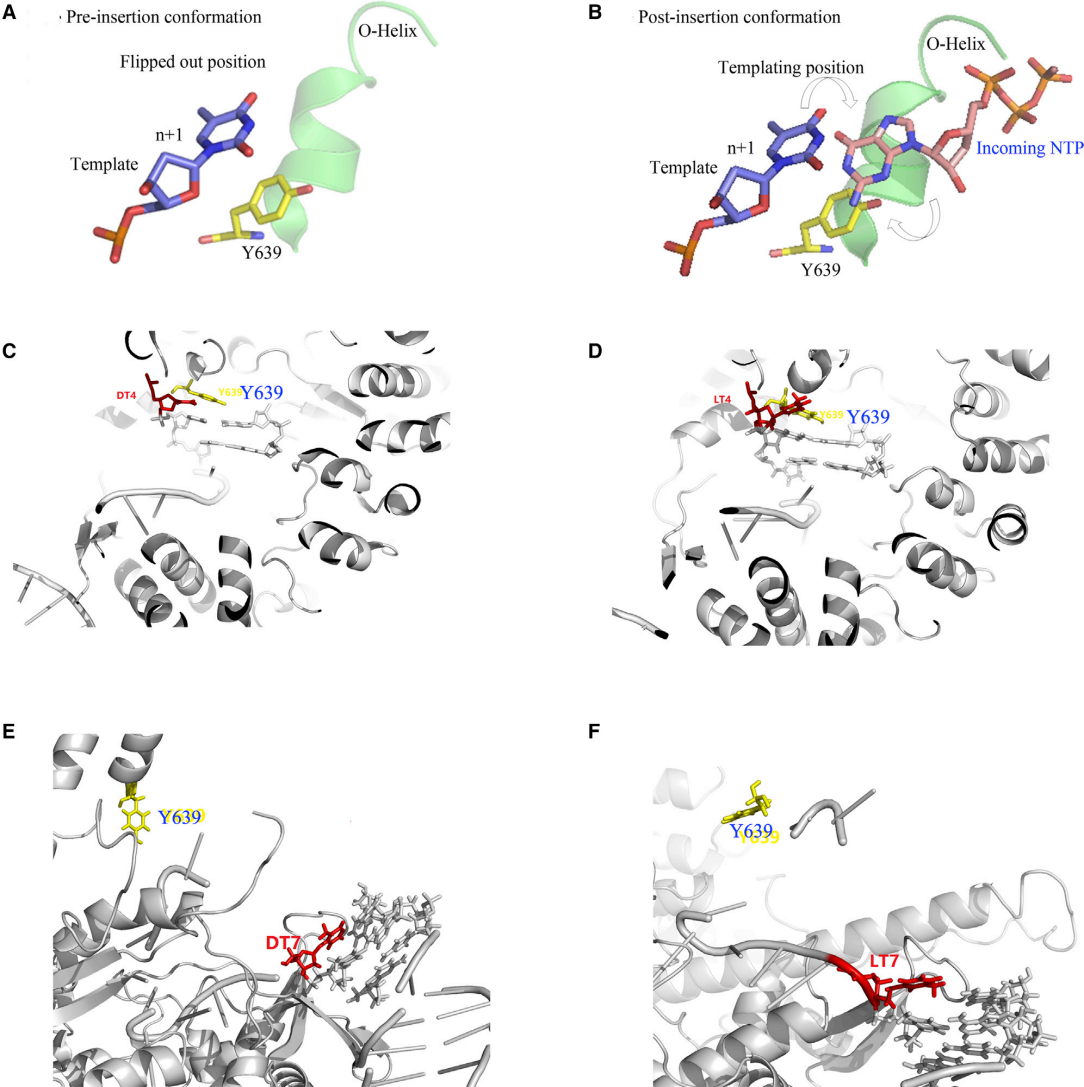
Insights into NTP Addition from 3D Structure of T7 RNAP (A–F) Conformation of the active center of T7 RNAP before (A) and after (B) NTP insertion or in complex with template T+4/N (C, d-T; D, l-T; PDB: 1QLN) or template T+7/N (E, d-T; F, l-T; PDB: 3E2E).

Extensive studies have been devoted to analysis of the 3D structure of the T7 RNAP transcribing or initiation complex. Thus, insights into NTP addition from 3D structure may provide useful information for understanding our results. T7 RNAP resembles a canonical “right hand” with thumb, palm, and fingers domains.^37^ The flexible fingers domains consist of an O helix with five α helices.^20^ The finger subdomain of the enzyme switches from open to closed to recruit an incoming NTP from a pre-insertion binding site in each nucleotide addition cycle (Figure S7).^18^ Moreover, residue Y639, which is adjacent to the O helix, has a critical role in RNA synthesis.^40^ It helps capture and incorporate incoming NTP by moving out of the active site to allow the insertion of a NTP in nucleotide addition cycle (Figures 9A and 9B).^18^ The incoming NTP makes hydrogen-bonding contacts with the templating base prior to entering the active site.^18^

The schematic illustration of the active site of the transcriptional complex gives us a comprehensive understanding of the NTP binding process. As shown in Figures 9C and 9D, with template T+4/N (d-T) or T+4/N (l-T) as template, Y639 is able to contact with the complex in the entrance and induce conformational changes of ATP to obtain a correct orientation for base pairing. However, ATP at pre-insertion associates closely with Y639 for template T+7/N (d-T) (Figure 9E) but stays far from the template T+7/N (l-T) (Figure 9F). Consequently, the incorrect NTP is caught by Y639 with T+7/N (l-T) as template, which is unlikely to be further inserted. Thus, an off-path pre-insertion configuration sterically blocks the rotation of Y639 to some extent and prohibits it from nucleotide insertion. No significant change in K_m_ and an obvious decrease in k_cat_ for T+4/N indicate that only the rate of product formation was decreased (Table 1). This may be explained by the fact that only three base pairs of the hetero-duplex are formed before the RNA peels off the template during accumulation or “scrunching” of the template in the enclosed active site pocket of T7 RNAP in the initiation phase.^41^ K_m_ for T+7/N was slightly increased and k_cat_ for T+7/N was decreased, indicating that affinity of RNAP and formation rate of transcripts were decreased after the introduction of l-T into position +7 of the transcribed region (Tables 1 and S2). This may be explained by the fact that steric hindrance of l-T resulted in a decreased rate of elongation and increased time required to promoter dissociation and RNA formation during synthesis. Thus, insertion of incoming NTP becomes relatively difficult for template T+7/N. In contrast, when the length of transcripts is greater than 8–12 nt, T7 RNAP releases the promoter sequence and enters an elongation mode. Transcription reaction is highly efficient in this phase. When l-T is introduced into position +8, +10, or +12 of the transcribed region (T+8/N, T+10/N, and T+12/N), the slight steric constraint does not hinder the rotation of Y639 and the release of promoter. In consequence, there is no remarkable alteration in the transcription efficiencies of template T+8/N, T+10/N, and T+12/N (Table S2).

Since the l-T and d-T have identical functional groups, substitution of d-T with l-T in the promoter region (T−3/N, T−10/N, T/N−2, and T/N−8) and non-template strand (T/N+11) causes relatively small steric hindrance when T7 RNAP binds to the promoter. With no significant steric clashes, introduction of l-T into the promoter region (T−3/N, T−10/N, T/N−2, and T/N−8) and non-template strand (T/N+11) does not much affect the binding or dissociation of T7 RNAP from the promoter. Thus, transcription efficiencies had no significant changes compared with normal template (Tables S3 and S4). Introduction of two consecutive l-Ts into the transcribed region (T+7+8/N) significantly disorders the template duplex, and thus binding of RNAP will be strongly interfered with because of its strict recognition. In addition, due to the accumulated steric hindrance, the rotation of Y639 is suppressed. In this case, insertion of NTP is sterically impossible, and thus transcription would be strongly interfered with (Figure S5). In addition, the accumulated steric clash may hamper the release of enzyme from the promoter, which also hinders the elongation of transcripts. Altogether, transcription of templates containing two consecutive l-Ts is strongly hampered. Distinguishing between correct and incorrect nucleotides of T7 RNAP is of great significance for its high efficiency and fidelity regulation of transcription. The newly transcribed RNA chain forms a DNA-RNA hybrid duplex inside the pocket.^34^^,^^40^ Consequently, only a correct Waston-Crick base pair will fit into the pocket.^24^ The rigid active site cannot accommodate the various base pairs equally well.^43^ The incorrect one will be withdrawn from the active site and rejected back to solution.^24^ Y639 of T7 RNAP occupies the insertion site, thereby allowing the insertion of matched base pairs and blocking the unmatched ones.^44^ Therefore, only ATP is incorporated with the complementary nucleotides (d-T or l-T) on the template.

## Materials and Methods

### Materials and Reagents

T7 RNAP was purchased from Beijing Biolink Biotechnology (NEB, Beijing, China). RNase inhibitor was bought from Vazyme Biotech. The NTP mixtures were from Shenggong Co. (Beijing, China). DTT was obtained from Solarbio Life Sciences, and l-T was from Hanwei BioTechnologies (AnhuiWuhu, China). Normal deoxynucleotide phosphoramidites for automated DNA synthesis were obtained from Anhui WuhuHuaren Biotechnique.

### Synthesis and Purification of l-T

As shown in Figure S1, l-T phosphoramidite was synthesized from l-T via 4,4'-dimethoxytrityl (DMT) deprotection of one hydroxyl group and following the reaction of the other hydroxyl group with phosphine reagent, and ^1^H nuclear magnetic resonance (NMR), ^13^C NMR, and ^31^P NMR (Figures S2–S4) were utilized to characterize its structure. l-T phosphoramidite was introduced to the oligonucleotides using a 3400 DNA synthesizer (Applied Biosystems) with standard reagents and program. After incubation with concentrated ammonium hydroxide for 10 h at 50°C, the oligonucleotides were cleaved from solid supports. Then, the oligonucleotides were purified by reversed phase high-pressure liquid chromatography (RP-HPLC) with a Waters reverse phase C_18_ column (5-μm bead, 9.6 × 250 mm). Oligonucleotide concentrations were determined by UV absorbance at 260 nm using a NanoDrop spectrophotometer (Thermo Scientific). Electrospray ionization mass spectrometry (ESI-MS) was used to identify the oligonucleotides (Table S1; Figure S5).

### Transcription Reaction by T7 RNAP

Double-stranded DNA templates were prepared by independently combining equimolar ratios of template and non-template strands in 10 mM NaCl, 40 mM Tris (pH 7.8), 6 mM MgCl_2_, 2 mM spermidine, and 10 mM DTT, annealing at 95°C for 5 min. The reaction mixtures were prepared in a total volume of 20 μL with 10 mM NaCl, 40 mM Tris (pH 7.8), 6 mM MgCl_2_, 2 mM spermidine, 1 μM DNA template, 1 mM NTP mixtures, 5 mM DTT, and 1 U/μL RNA inhibitor. After the addition of T7 RNAP (50 U/μL), the reaction mixtures were gently vortexed and incubated at 37°C for 4 h to achieve transcripts. Then, the mixtures were mixed with an equal volume of 2× loading buffer (95% formamide, 25 mM EDTA, 0.02% xylene cyanol, and 0.02% bromophenol blue) and incubated at 90°C for 5 min. A 10-μL aliquot of the mixture was subjected to electrophoresis on a 20% denaturing polyacrylamide gel containing 7 M urea at 100 V for 2 h in the presence of a 1× Tris/borate/EDTA (TBE) buffer. DNA templates and RNA products separated on the gel were stained with SYBR Gold (L/N S11494, Invitrogen) and visualized by the ChemiDoc XRS system (Bio-Rad).

### Transcription Kinetics Analysis

For single time points, the transcription reactions were done as described above. The reaction mixtures were incubated for different time intervals (1, 5, 10, 20, 40, 60, 80, 100, and 120 min) and stopped with an equal volume of 2× loading buffer (95% formamide, 25 mM EDTA, 0.02% xylene cyanol, and 0.02% bromophenol blue). After incubation at 90°C for 5 min, products of transcription were resolved by denaturing gel electrophoresis (20% polyacrylamide, 7 M urea) and visualized by the ChemiDoc XRS system (Bio-Rad). Quantitative analysis of the running start assays was performed by determining the relative lesion bypass efficiencies (l-T triphosphate bypass %) as a function of reaction time. All reactions were performed in triplicate.

For the determination of kinetic parameters, transcription was carried out by changing the concentration of NTP mixtures according to previous reports.^31^^,^^34^ A total volume of 20 μL containing 10 mM NaCl, 40 mM Tris (pH 7.8), 6 mM MgCl_2_, 2 mM spermidine, 1 μM DNA template, NTP mixtures varying from 0 to 1.0 mM, 5 mM DTT, and 1 U/μL RNA inhibitor was prepared. Reactions were initiated by addition of 2 μL (50 U/μL) of polymerase and were run at 37°C. Aliquots (10 μL) were taken at 4 h, mixed with an equal volume of 2× loading buffer used above, and electrophoresed on 20% denaturing polyacrylamide gel containing 7 M urea. Transcripts bands were quantitated using the ChemiDoc XRS system (Bio-Rad). Percent incorporation was translated into the rate of transcript synthesis as a function of [NTP], and rate data were fit by nonlinear least squares using the Michaelis-Menten equation to derive K_m_ and k_cat_ values.

### Mismatch Analysis

The mismatch was analyzed by a gel fidelity assay. After annealing at 95°C for 5 min, 2 μL of template-non-template complexes (10 μM), 2 μL of RNAP reaction buffer, 2 μL of T7 RNAP (50 U/μL), 2 μL of 50 mM DTT, 4 μL of GTP (2.5 mM), and 4 μL of diethyl pyrocarbonate (DEPC) water were mixed. Transcription was initiated by adding 4 μL of 2.5 mM CTP, ATP, or UTP, respectively. The mixtures were incubated at 37°C for 8 h. The polymerase reactions were quenched by the addition of 20 μL of loading buffer (95% formamide, 0.05% bromophenol blue, 0.05% xylene cyanol, and 20 mM EDTA). After heating at 90°C for 5 min, the products were analyzed as described above.

## Author Contributions

Q.L. performed the experiments and wrote the manuscript. Y. Ke helped with statistical analysis. Y.Kan helped conduct the gel fidelity analysis. X.T. helped with the synthesis of templates. X.L. helped analyze the data. Y.H. helped to conceive the research. L.W. conceived the research, analyze the data, and corrected the manuscript.

## Conflicts of Interest

The authors declare no competing interests.

## Supplemental Information

Document S1. Figures S1–S7, Tables S1–S6, and Supplemental Materials and Methods

Document S2. Article plus Supplemental Information
