# Supplementary material for: Compatibility and Fidelity of Mirror-Image Thymidine in Transcription Events by T7 RNA Polymerase
Source: Mol Ther Nucleic Acids. 2020 Jun 27;21:604–13. doi: 10.1016/j.omtn.2020.06.023 (PMC7390857; doi:10.1016/j.omtn.2020.06.023)
Supplement: Document S1. Figures S1–S7, Tables S1–S6, and Supplemental Materials and Methods [file mmc1.pdf]

**OMTN, Volume *21***

## Supplemental Information

# Compatibility and Fidelity of Mirror-Image Thymidine in Transcription Events by T7 RNA Polymerase

**Qingju Liu, Yongqi Ke, Yuhe Kan, Xinjing Tang, Xiangjun Li, Yujian He, and Li Wu**

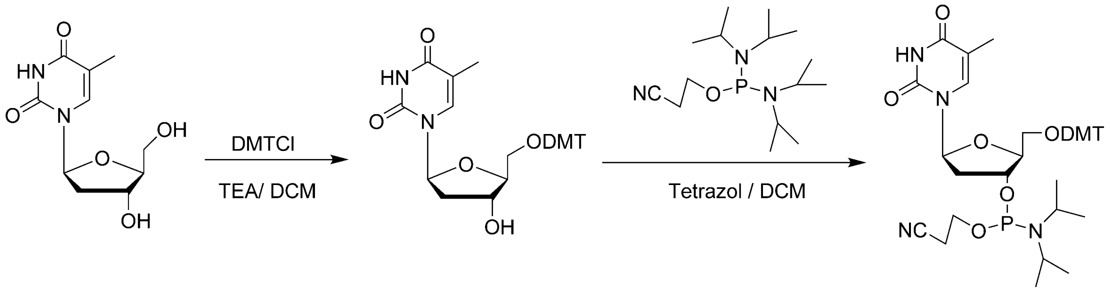


**Figure S1** Synthetic process of L-thymidine phosphoramidite

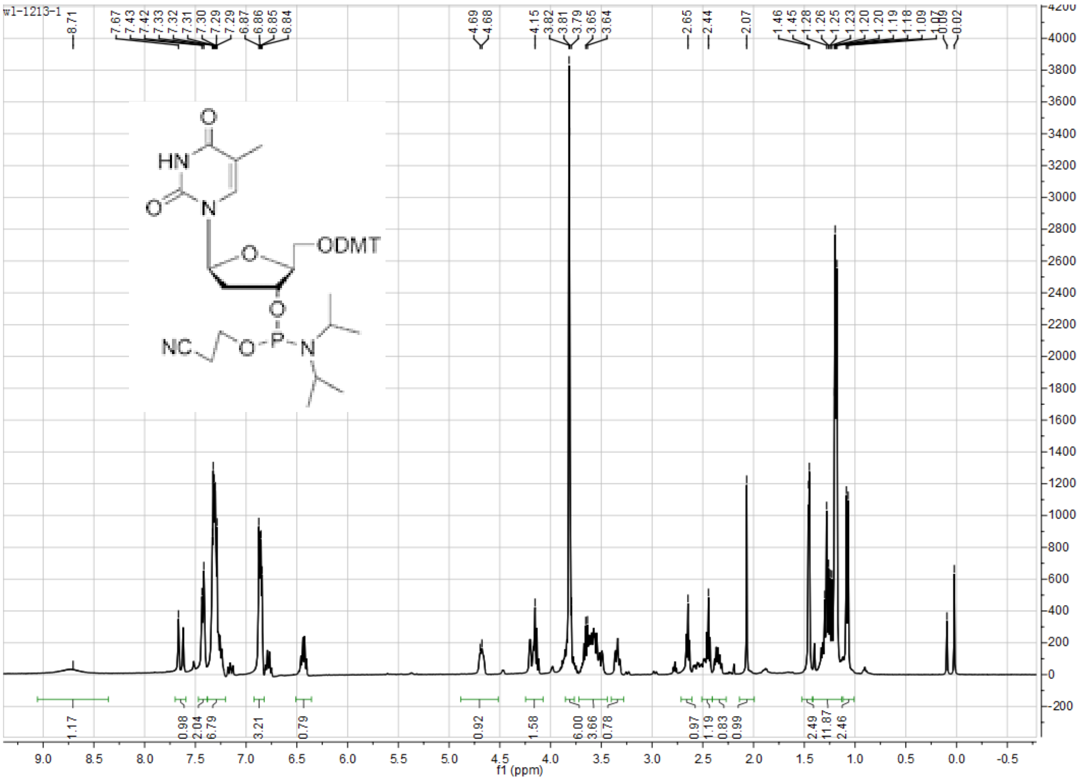


**Figure S2** $^{1}$H NMR of L-thymidine phosphoramidite

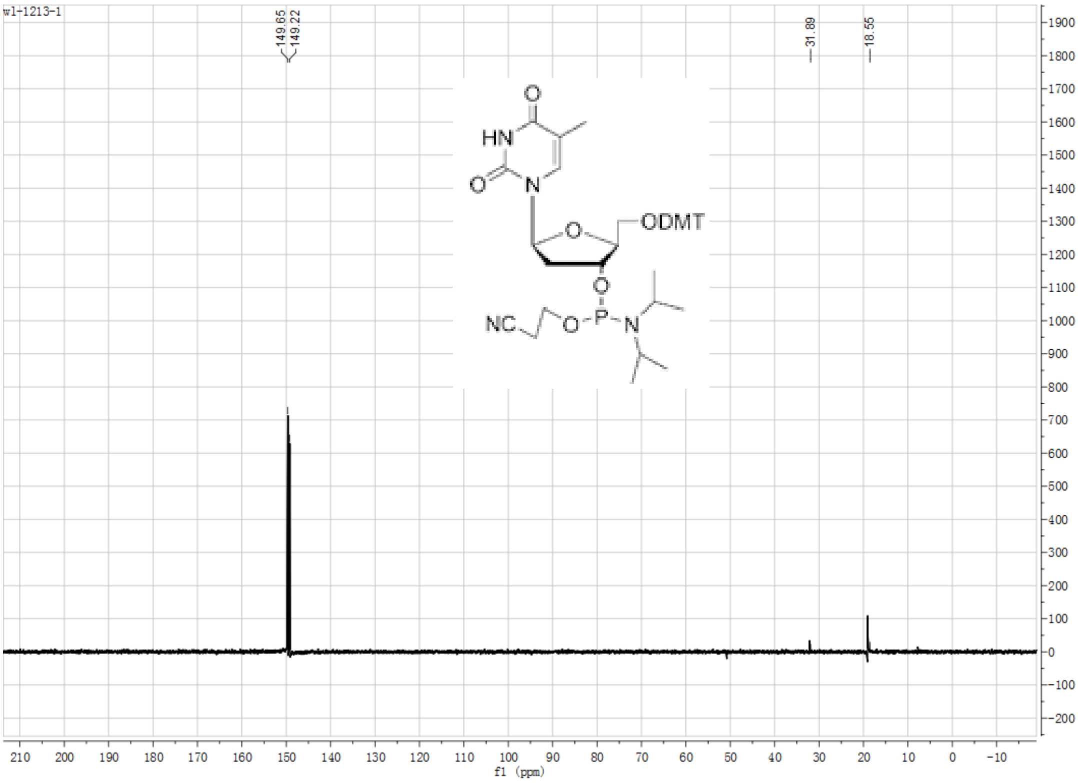

**Figure S3** $^{31}$P NMR of L-thymidine phosphoramidite

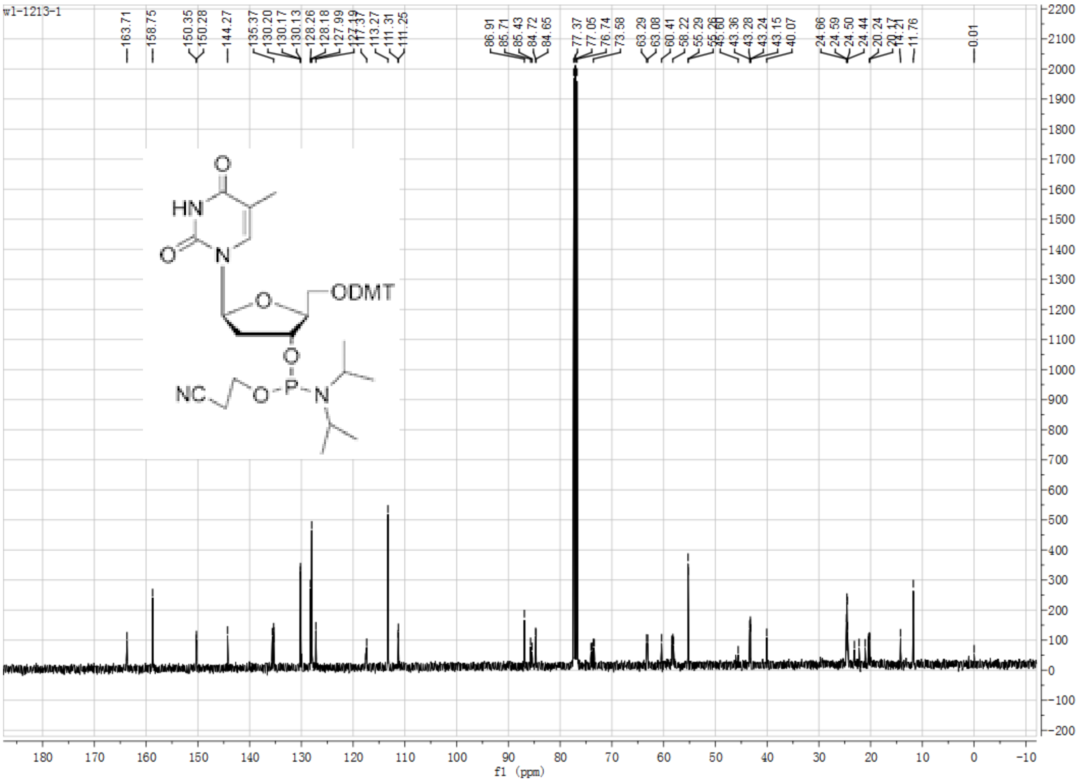

**Figure S4** $^{13}$C NMR of L-thymidine phosphoramidite

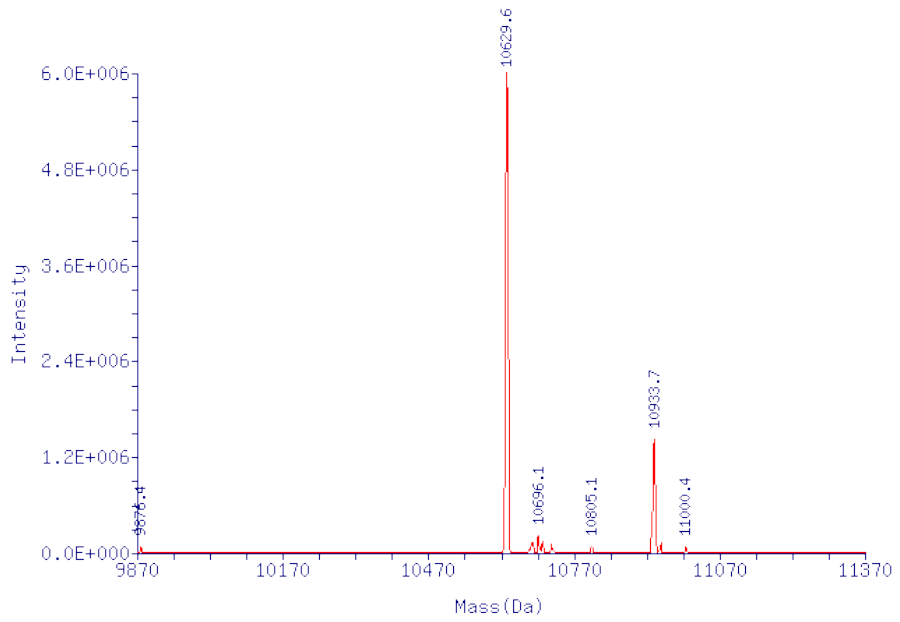
10629.6
6.0E+006
4.8E+006
3.6E+006
2.4E+006
1.2E+006
0.0E+000
Intensity
9876.4
10696.1
10805.1
10933.7
11000.4
9870
10170
10470
10770
11070
11370
Mass(Da)

T

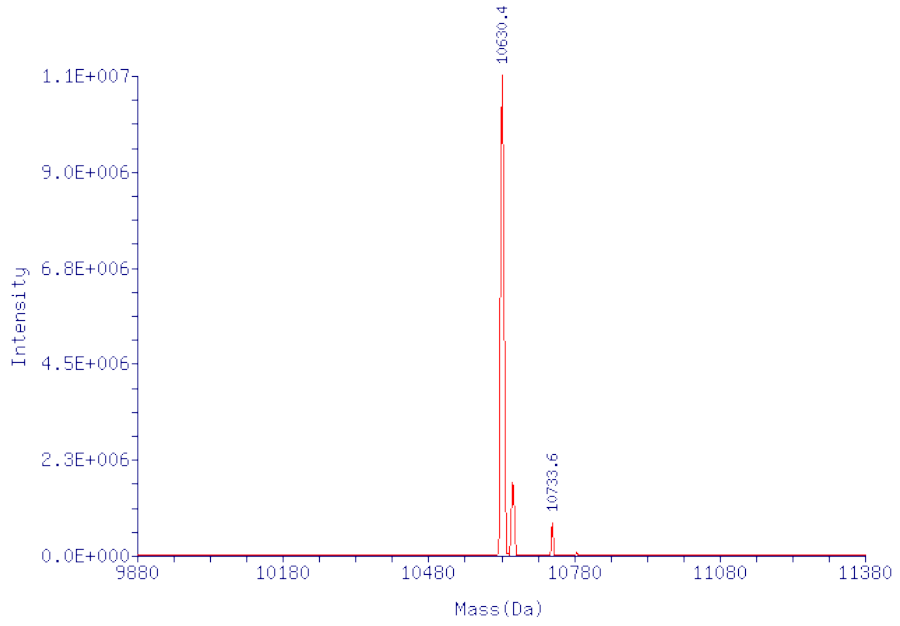
10630.4
1.1E+007
9.0E+006
6.8E+006
4.5E+006
2.3E+006
0.0E+000
Intensity
10733.6
9880
10180
10480
10780
11080
11380
Mass(Da)

T+4

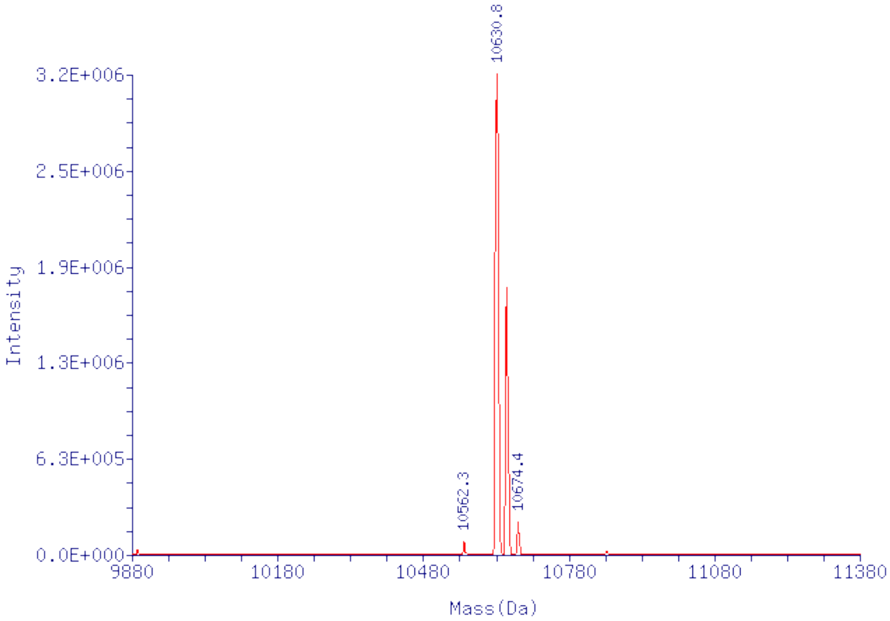
10630.8
3.2E+006
2.5E+006
1.9E+006
1.3E+006
6.3E+005
0.0E+000
Intensity
10562.3
10674.4
9880
10180
10480
10780
11080
11380
Mass(Da)

T+7

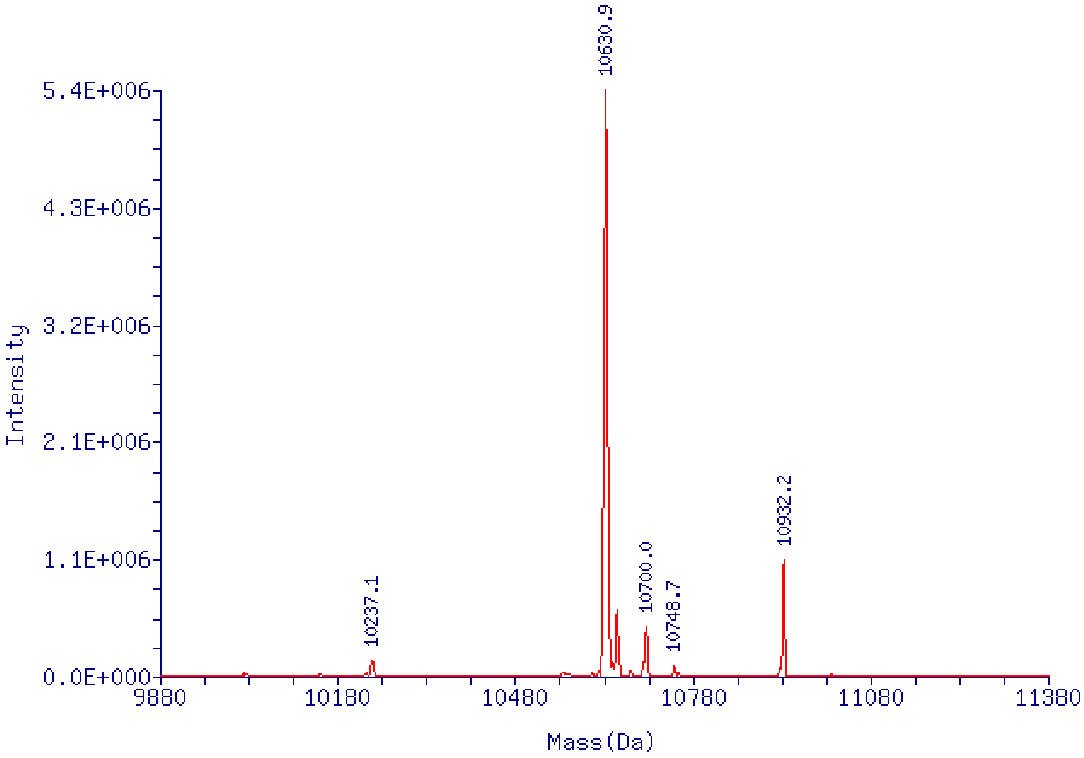
10630.9
5.4E+006
4.3E+006
3.2E+006
2.1E+006
1.1E+006
0.0E+000
Intensity
10237.1
10700.0
10748.7
10932.2
9880
10180
10480
10780
11080
11380
Mass(Da)

T+8

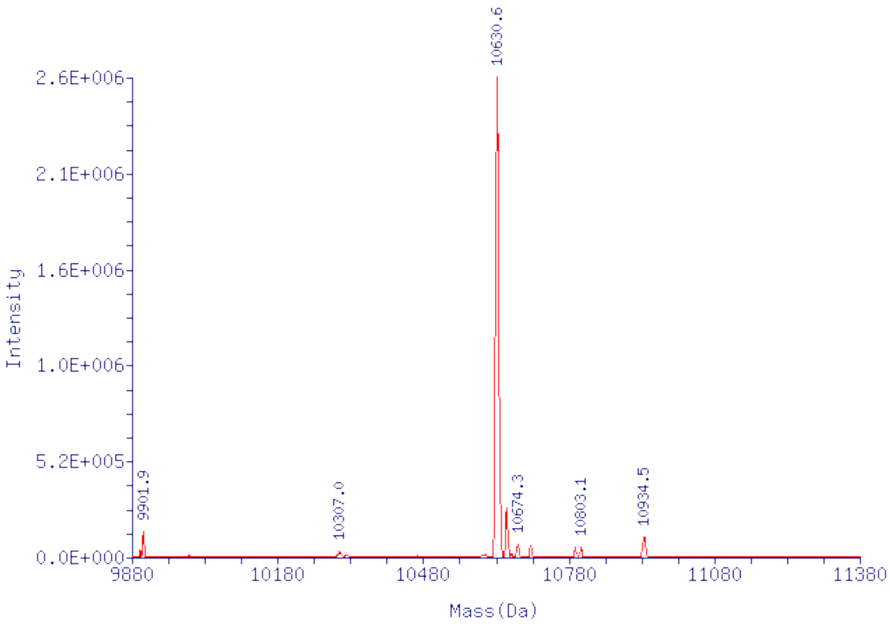
10630.6
2.6E+006
2.1E+006
1.6E+006
1.0E+006
5.2E+005
0.0E+000
Intensity
9901.9
10307.0
10674.3
10803.1
10934.5
9880
10180
10480
10780
11080
11380
Mass(Da)

T+7+8

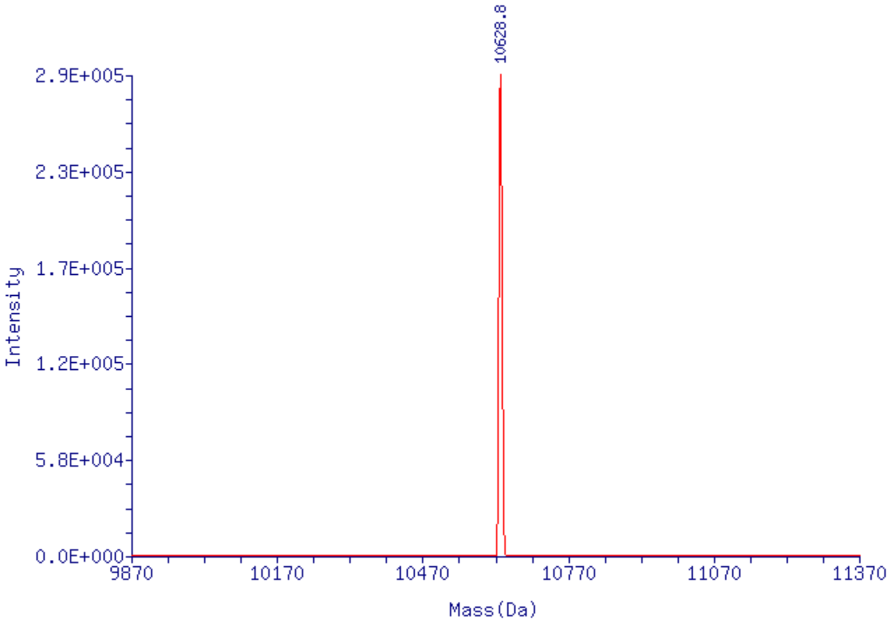
10628.8
2.9E+005
2.3E+005
1.7E+005
1.2E+005
5.8E+004
0.0E+000
Intensity
9870
10170
10470
10770
11070
11370
Mass(Da)

T+8+10

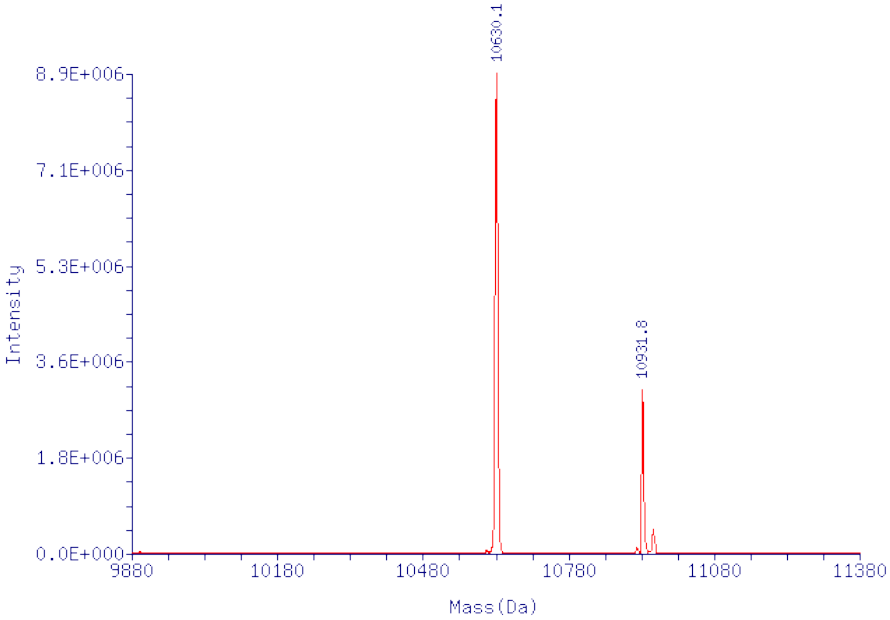
10630.1
10931.8
8.9E+006
7.1E+006
5.3E+006
3.6E+006
1.8E+006
0.0E+000
Intensity
9880
10180
10480
10780
11080
11380
Mass(Da)

T+10

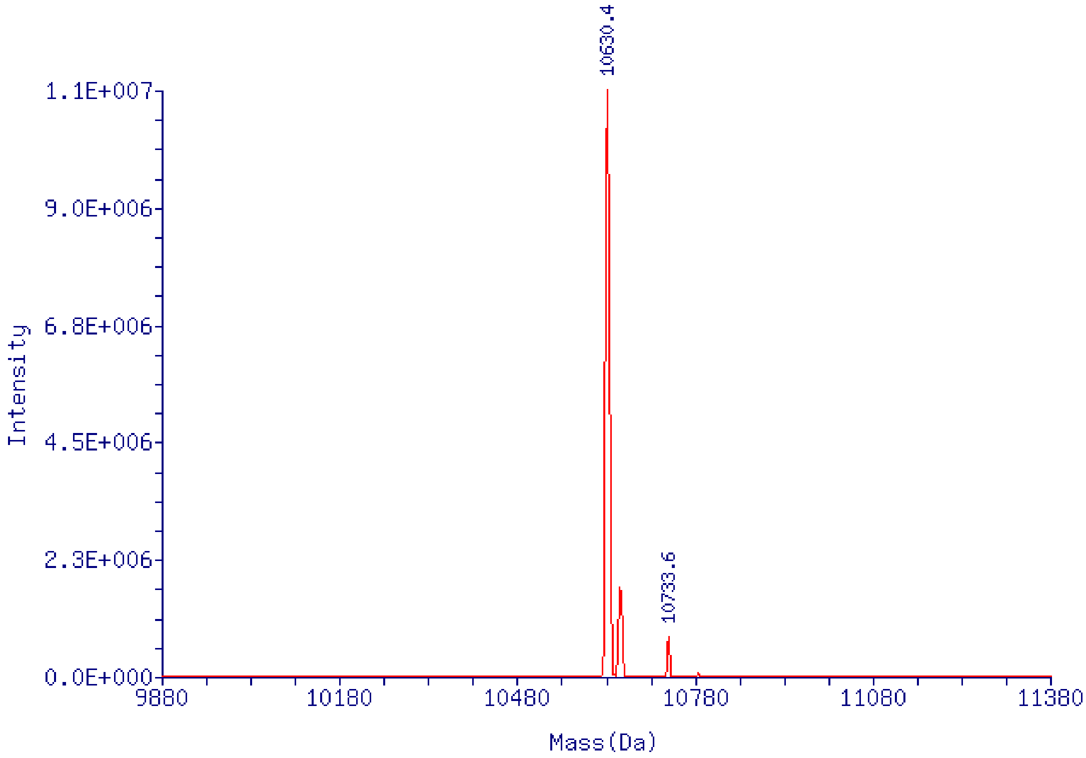
10630.4
10733.6
1.1E+007
9.0E+006
6.8E+006
4.5E+006
2.3E+006
0.0E+000
Intensity
9880
10180
10480
10780
11080
11380
Mass(Da)

T+12

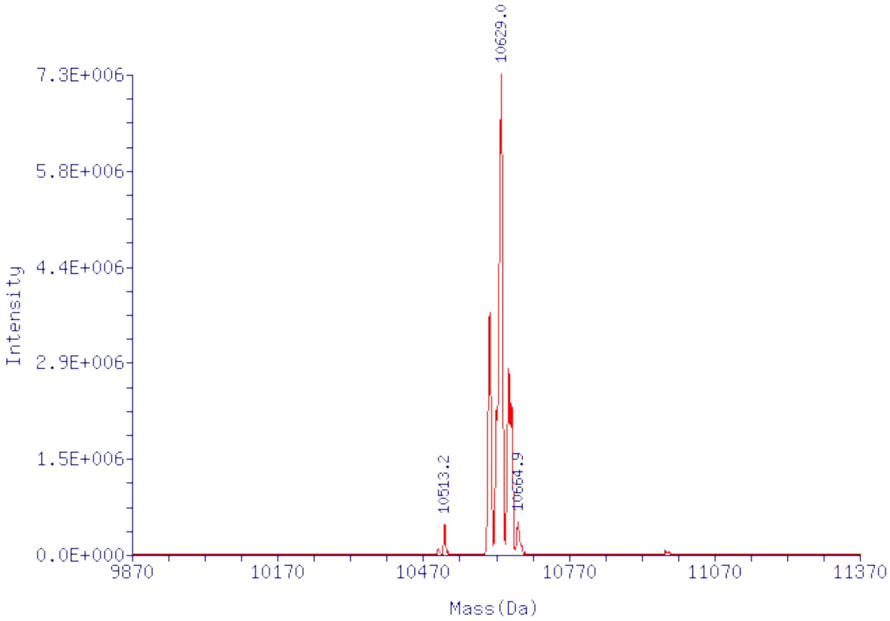
10629.0
7.3E+006
5.8E+006
4.4E+006
2.9E+006
1.5E+006
0.0E+000
Intensity
10513.2
10664.9
9870
10170
10470
10770
11070
11370
Mass(Da)

T-3

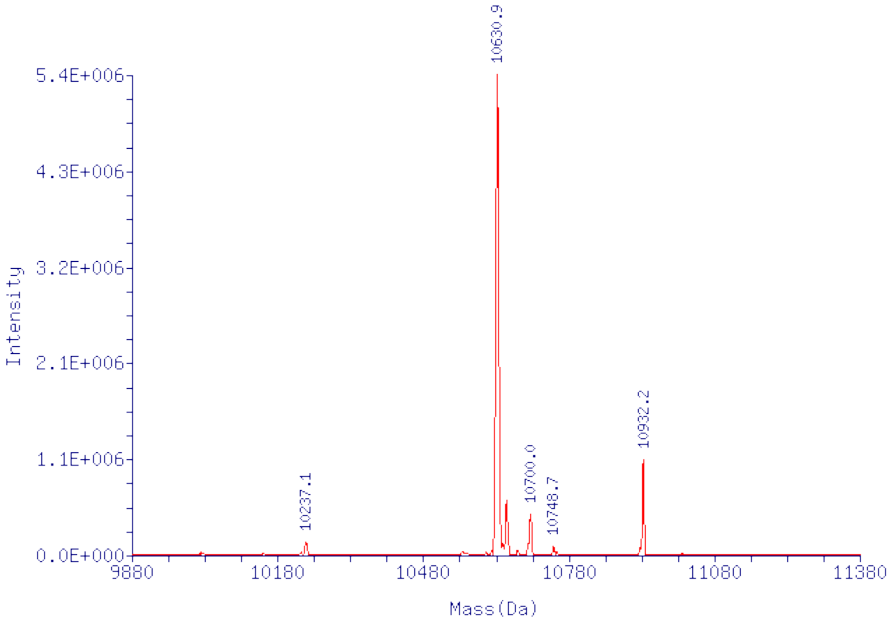
10630.9
5.4E+006
4.3E+006
3.2E+006
2.1E+006
1.1E+006
0.0E+000
Intensity
10237.1
10700.0
10748.7
10932.2
9880
10180
10480
10780
11080
11380
Mass(Da)

T-10

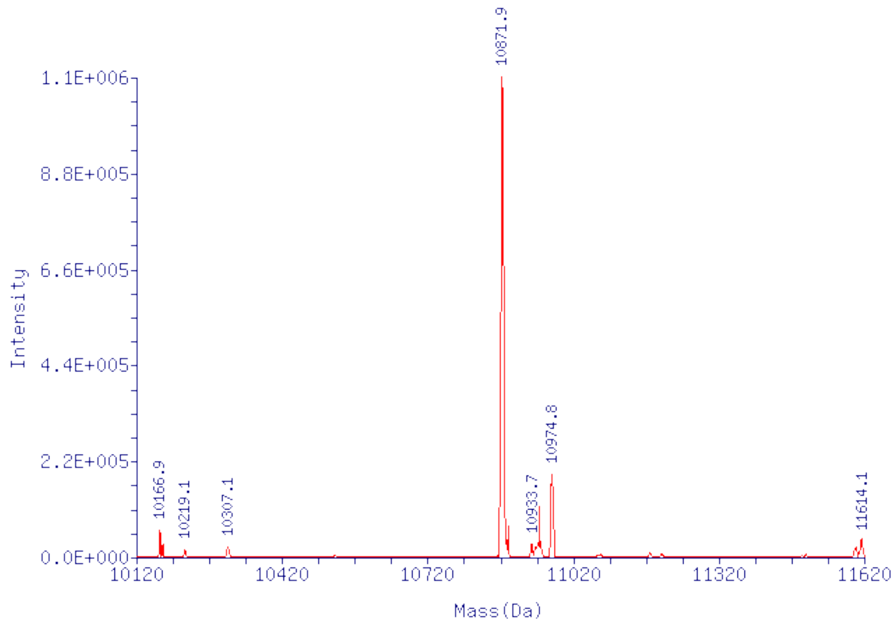
10871.9
1.1E+006
8.8E+005
6.6E+005
4.4E+005
2.2E+005
0.0E+000
Intensity
10166.9
10219.1
10307.1
10933.7
10974.8
11614.1
10120
10420
10720
11020
11320
11620
Mass(Da)

N

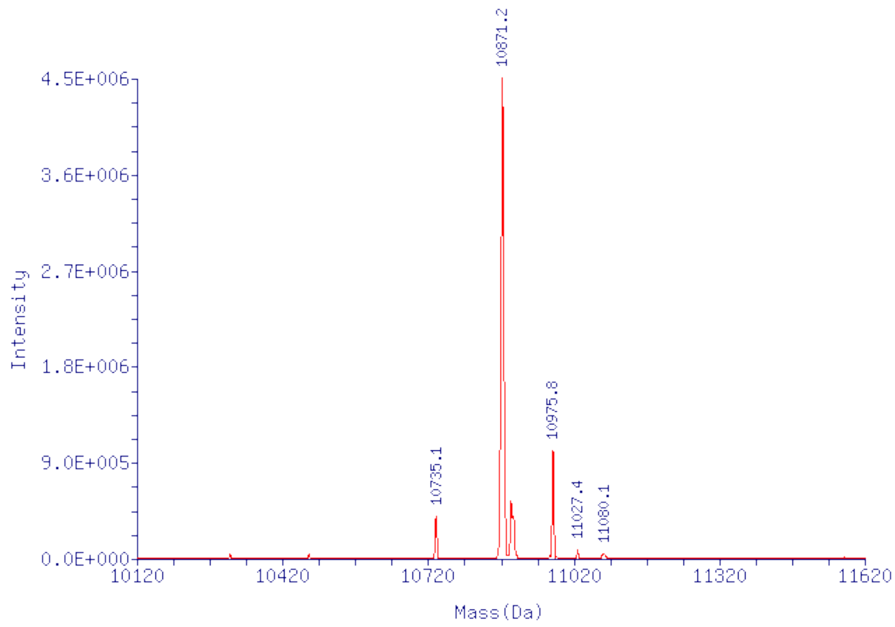
10871.2
4.5E+006
3.6E+006
2.7E+006
1.8E+006
9.0E+005
0.0E+000
Intensity
10735.1
10975.8
11027.4
11080.1
10120
10420
10720
11020
11320
11620
Mass(Da)

N-2

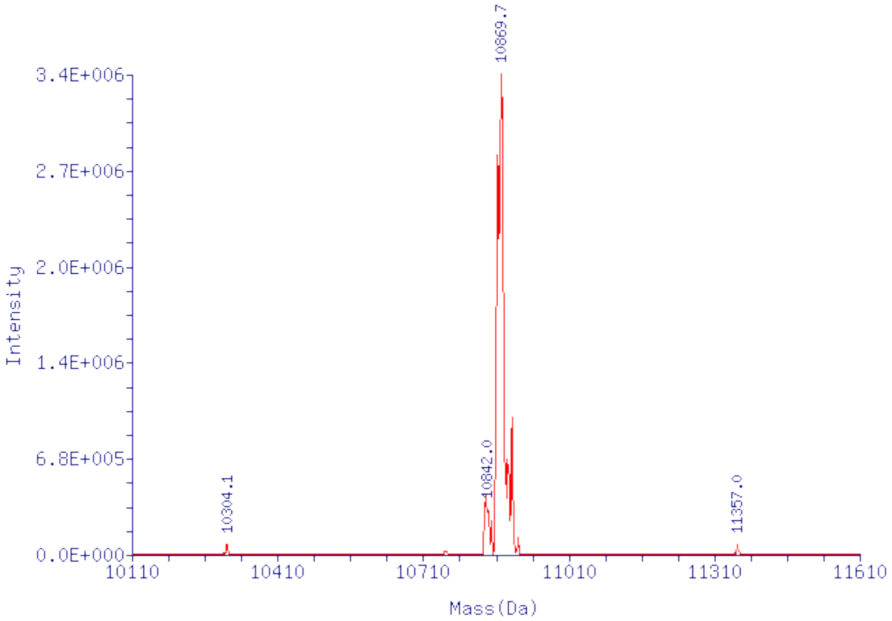


N-8

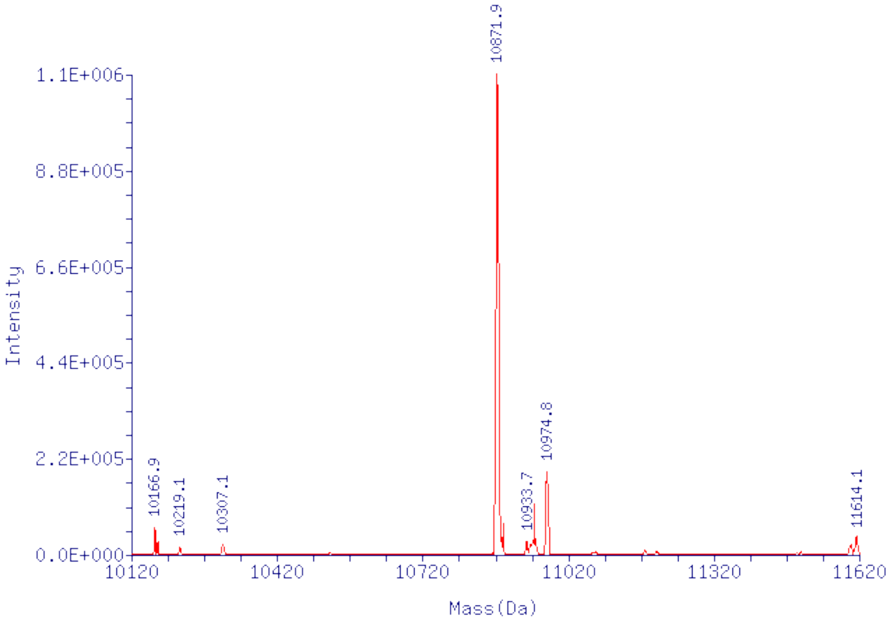


N+11

**Figure S5** ESI-MS of templates containing L-thymidine for transcription

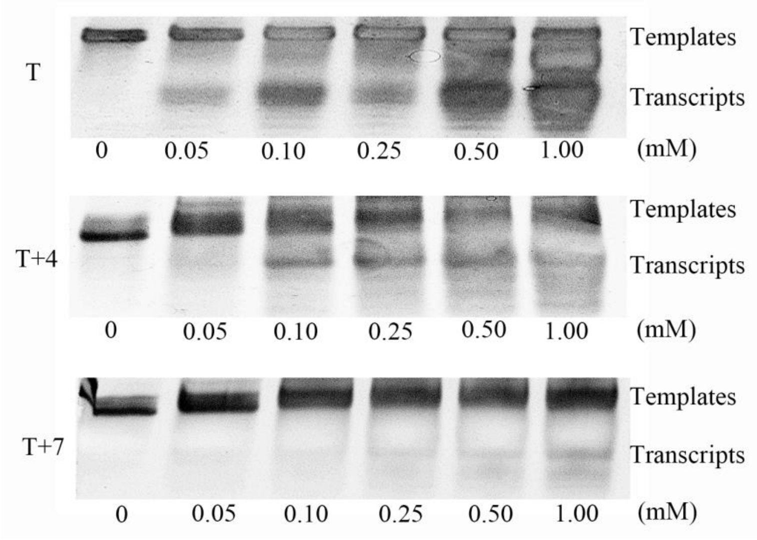


**Figure S6** Analysis of transcription kinetics of the three templates. The reaction mixtures were in a total volume of 20 μL containing 10 mM NaCl, 40 mM Tris (pH 7.8), 6 mM $MgCl_2$, 2 mM spermidine, 1 μM DNA template, NTP mixtures varying from 0 to 1.0 mM, 5 mM DTT and 1 U/μL RNA inhibitor. Reactions were initiated by addition of 2 μL (50 U/μL) polymerase and were incubated at 37 °C for 4 hours.

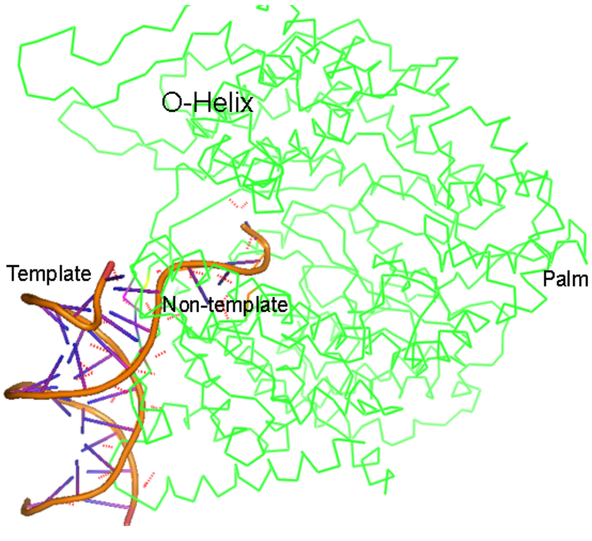


**Figure S7** Interaction between template and T7 RNA polymerase reported by Steitz et al [1] ( PDB ID: 1CEZ).

**Table S1** ESI-MS of templates containing L-thymidine

| Name | Theoretical value | Measured value |
|---|---|---|
| T | 10628.97 | 10629.6 |
| T+4 | 10628.97 | 10630.1 |
| T+7 | 10628.97 | 10630.8 |
| T+8 | 10628.97 | 10630.9 |
| T+7+8 | 10628.97 | 10630.6 |
| T+8+10 | 10628.97 | 10628.8 |
| T+10 | 10628.97 | 10630.1 |
| T+12 | 10628.97 | 10630.4 |
| T-3 | 10628.97 | 10629 |
| T-10 | 10628.97 | 10630.9 |
| N | 10870.18 | 10871.9 |
| N-2 | 10870.18 | 10871.2 |
| N-8 | 10870.18 | 10869.7 |
| N+11 | 10870.18 | 10871.9 |

**Table S2** Bypass of transcripts from templates containing L-T at different positions of transcribed region

| Template | Amount of enzyme (U) | Bypass of aborted products (%) | Bypass of full-length products (%) |
|---|---|---|---|
| T/N | 0 | 0.0 | 0.0 |
| | 25 | 0.0 | 4.8 |
| | 50 | 0.0 | 18.9 |
| | 100 | 0.0 | 35.4 |
| T+4/N | 0 | 0.0 | 0.0 |
| | 25 | 0.0 | 2.1 |
| | 50 | 0.0 | 12.2 |
| | 100 | 0.0 | 34.0 |
| T+7/N | 0 | 0.0 | 0.0 |
| | 25 | 1.4 | 1.8 |
| | 50 | 4.8 | 3.8 |
| | 100 | 13.5 | 9.5 |
| T+8/N | 0 | 0.0 | 0.0 |
| | 25 | 0.0 | 3.9 |
| | 50 | 0.0 | 17.2 |
| | 100 | 0.0 | 35.6 |
| T+10/N | 0 | 0.0 | 0.0 |
| | 25 | 0.0 | 8.9 |
| | 50 | 0.0 | 17.3 |
| | 100 | 0.0 | 39.0 |
| T+12/N | 0 | 0.0 | 0.0 |
| | 25 | 0.0 | 3.0 |
| | 50 | 0.0 | 14.7 |
| | 100 | 0.0 | 36.8 |

**Table S3** Bypass of transcripts from templates containing L-T at different positions of non-transcribed region in template strand

| Template | Amount of enzyme (U) | Bypass of full-length products (%) |
|---|---|---|
| T/N | 0 | 0.0 |
| | 25 | 4.5 |
| | 50 | 15.9 |
| | 100 | 35.4 |
| T-3/N | 0 | 0.0 |
| | 25 | 4.0 |
| | 50 | 12.2 |
| | 100 | 32.9 |
| T-10/N | 0 | 0.0 |
| | 25 | 4.0 |
| | 50 | 14.4 |
| | 100 | 33.8 |

**Table S4** Bypass of transcripts from templates containing L-T at different positions of non-template strand

| Template | Amount of enzyme (U) | Bypass of full-length products (%) |
|---|---|---|
| T/N | 0 | 0.0 |
| | 25 | 3.7 |
| | 50 | 11.7 |
| | 100 | 34.9 |
| T/N-2 | 0 | 0.0 |
| | 25 | 4.0 |
| | 50 | 12.3 |
| | 100 | 28.2 |
| T/N-8 | 0 | 0.0 |
| | 25 | 4.9 |
| | 50 | 17.3 |
| | 100 | 29.8 |
| T/N+11 | 0 | 0.0 |
| | 25 | 4.2 |
| | 50 | 15.8 |
| | 100 | 27.6 |

**Table S5** Bypass of transcripts from templates containing two L-Ts at different positions of non-template strand

| Template | Amount of enzyme (U) | Bypass of aborted products (%) | Bypass of full-length products (%) |
|---|---|---|---|
| T/N | 0 | 0 | 0.0 |
| | 25 | 0 | 4.5 |
| | 50 | 0 | 15.9 |
| | 100 | 0 | 35.4 |
| T+7+8/N | 0 | 0 | 0.0 |
| | 25 | 0.2 | 0.1 |
| | 50 | 0.6 | 0.2 |
| | 100 | 0.9 | 0.3 |
| T+10+12/N | 0 | 0 | 0.0 |
| | 25 | 0.2 | 0.1 |
| | 50 | 0.3 | 0.3 |
| | 100 | 0.5 | 0.3 |

**Table S6** Bypass of transcripts from natural template ($T_2/N_2$) and ($T_2$+7/$N_2$) in a transcription reaction catalyzed by T7 RNAP

| Template | Substrate | Bypass of band (6 nt) | Bypass of band (13 nt) | Percentage of band (6 nt, %) | Percentage of band (13 nt, %) |
|---|---|---|---|---|---|
| $T_2/N_2$ | G | 35.9 | 0 | 100 | 0 |
| | G+C | 41.7 | 0 | 100 | 0 |
| | G+U | 43.2 | 0 | 100 | 0 |
| | G+A | 4.4 | 30 | 12.8 | 87.2 |
| $T_2$+7/N2 | G | 43 | 0 | 100 | 0 |
| | G+C | 42.9 | 0 | 100 | 0 |
| | G+U | 43.6 | 0 | 100 | 0 |
| | G+A | 24.9 | 31.6 | 44 | 56 |

**MD simulation**

MD simulation was performed with AMBER 14 molecular package. We used Gaussian 09 program to optimize the GTP molecular in the complexes, and used amber force to handle the complexes of polymerases and nucleic acids. First, we get the D-structure from PDB database. After that, to obtain the L-nucleic acids , we rebuilt the force field about the L-nucleic acids , and then deleted other atoms in the DT residues except for main chain atoms which includes P , OP1 , OP2 , O5' , C5' , C4' , O4' , C3' , O3' . Through the L-nucleic acids force field, we obtained the complexes of polymerases and L-nucleic acids . Finally, the picture is performed with PYMOL.

1. Cheetham, G. M.; Jeruzalmi, D.; Steitz, T. A., Structural basis for initiation of transcription from an RNA polymerase-promoter complex. *Nature* **1999,** *399* (6731), 80-3.
